# Screen-time during the after-school period: A contextual perspective

**DOI:** 10.1016/j.pmedr.2020.101116

**Published:** 2020-05-08

**Authors:** Emma Haycraft, Lauren B. Sherar, Paula Griffiths, Stuart J.H. Biddle, Natalie Pearson

**Affiliations:** a National Centre for Sport and Exercise Medicine, School of Sport, Exercise & Health Sciences, Loughborough University, Loughborough, UK; b Institute for Resilient Regions, University of Southern Queensland, Springfield, Australia

**Keywords:** Sedentary behaviour, Television viewing, Screen-use, Adolescents, Behaviour change, Health promotion

## Abstract

Sedentary screen-time is an increasingly prevalent behaviour, associated with a range of adverse health outcomes. Sedentary time and screen-use increase during adolescence, making this age group a prime target for behaviour change interventions. Better understanding the context in which sedentary screen-behaviours occur is important for ensuring future interventions have maximum impact. This study aimed to describe the prevalence of adolescents’ sedentary screen-time in the after-school and weekday evening periods, and to examine associations between contextual factors (location within the home and who they were with) and after-school/evening screen-time. Time that UK adolescents (N = 204, aged 11 or 12 years, 61.4% girls) spent using various screens was measured using a detailed three-day time-use diary completed at home. Adolescents reported the start and end time for each screen-based activity, where they were, and who they were with. Weekday (Monday-Friday) data were analysed with a focus on the after-school (3–6 pm) and evening periods (6–10.45 pm). Young adolescents spend around a third of their weekday evening leisure-time using screens, with boys engaging in slightly more screen-use than girls. The majority of after-school and weekday evening time at home was spent with family or siblings, with less than 1% spent with friends. Adolescents who spent more time alone after school reported greater screen-use. Greater time spent at home, in the lounge (living room) or bedroom was associated with greater screen-use. These findings highlight the value of devising family-based health-promotion interventions which target after-school/leisure-time screen-use in an effort to reduce young adolescents’ sedentary recreational screen-time behaviours.

## Highlights

• A third of young adolescents’ time after school is spent using screens.
• Spending more time at home after school is linked with greater screen-use.
• Spending time alone after school is associated with greater screen-use.
• Interventions should focus on reducing leisure-time screen-use, especially at home.
• Family-based interventions should target adolescents’ time spent alone at home.

Sedentary behaviours are highly prevalent in young people and time spent sedentary is associated with adverse physical and psychosocial health and conditions like obesity, insulin resistance and depression (Carson et al., 2016, Liu et al., 2016, Boers et al., 2019). Use of screens (i.e. television viewing and/or computer/tablet/smartphone use) is the most prevalent leisure-time sedentary behaviour in young people, with substantial proportions exceeding the guideline of two hours/day of recreational screen-time (Ofcom, 2018, Thomas et al., 2019, Pearson et al., 2019). Recent evidence suggests that adolescents spend over 70% of their after school time sitting (Arundell et al., 2019), with around half of this time spent using screens (Arundell et al., 2016). Furthermore, screen-use increases as children transition to adolescence (Pearson et al., 2017), with one British study showing weekly screen-use to increase between age 10 and 13/14 from 8.1 to 15.2 h in boys and 6.1 to 15 h in girls (Atkin et al., 2013), and it tracks modestly into adulthood (Biddle et al., 2010) making this age-group a prime target for health promotion interventions.

Family TV-viewing, child age, ethnicity, socioeconomic position (SEP) and home environment (e.g., screen accessibility) are predictors of screen-use (LeBlanc et al., 2015, Tandon et al., 2012) and screen-use is linked to psychosocial health conditions like depression (Carson et al., 2016, Liu et al., 2016, Boers et al., 2019). Although research shows that the number of televisions and having a television in the bedroom (LeBlanc et al., 2015) are associated with increased television viewing, and that television viewing can displace other activities and social interactions (Vandewater et al., 2006), little research has documented the context in which various screen-time behaviours occur, specifically, **where** and **with whom** an adolescent is using screens. For example, little is known about whether adolescents use screens individually or with others (e.g., family/friends) and where this after school screen-time predominantly occurs (e.g., in the bedroom or lounge). This study therefore aimed to describe the prevalence of adolescents’ sedentary screen-time in the after-school and weekday evening periods and to examine associations between contextual factors (location within the home and who they were with) and adolescents’ after school screen-time.

## 1 Method

### 1.1 Participants

Young adolescents (N = 527; 11 or 12-years-old) participated from four UK secondary schools. All students in Year 7 (aged 11/12 and in their first year of high/secondary school) were eligible and received an information leaflet to take home for a parent/guardian. Only adolescents with parental consent and individual assent, and who completed at least one diary entry, are reported on here (n = 204; response rate 39%^1^).

### 1.2 Measures and procedure

Following institutional ethical approval, adolescents completed questionnaires during a school lesson, supervised by trained researchers. A time-use diary was given for completion at home.

#### 1.2.1 Demographic information

Adolescents reported their age, sex, ethnicity and the number of adults who resided at their home. They also provided their home postcode which was used to determine community SEP using the Index of Multiple Deprivation (IMD). Adolescents were coded as ‘low’, ‘middle’, or ‘high’ SEP based on their IMD.

#### 1.2.2 Time-use diary

Time that adolescents spent using various screens was measured using a detailed time-use diary. The diary is based on Ecological Momentary Assessment (Dunton et al., 2005) principles and is a valid, reliable tool for assessing time allocations to sedentary behaviour and physical activity in secondary school children (Gorely et al., 2007). Adolescents were asked to complete a time-use diary on two school days and one weekend day over a week. Parents were asked to remind their child to complete the diary and to help if required. For the present study, only weekday data were analysed as the focus was on the after-school period. Data collection days were randomly assigned at the school level. On school days, time-use diaries were split into 15-minute intervals from 3 pm to 10.45 pm and adolescents were asked to report the time that they started and stopped each screen-based activity, and what they were doing (e.g. ‘what is the main thing you are doing?’). To aid accurate completion, examples common to young people were provided (e.g. talking with friends, watching TV, walking to school, etc.). Adolescents also responded to two open-response items for each time period: where they were (location) and who they were with.

For each data collection day, 33 time-samples were obtained (one every 15 min from 3 pm to 10.45 pm). For this study, the after-school period was classified as the time between 3 pm and 6 pm, and the evening period was the time between 6 pm and 10.45 pm.

### 1.3 Diary synthesis and coding

Given this study’s focus, only screen-based behaviours were analysed. Screen-time reports were coded into four mutually exclusive categories of leisure-time screen-based behaviour (watching TV/DVDs; using a computer/internet; playing computer/video games; and using a smartphone/tablet). These categories were then coded into one ‘screen-time’ category. To estimate the time spent in this screen-time category, interval-level school day data were aggregated for each individual by multiplying the daily frequency of the event by 15 (1 interval = 15 min). Data were then aggregated further to produce a mean, in minutes/day, across days. Data were reduced to mean minutes per after school and evening period spent in screen-time activity (the primary variable for this analysis). Proportions of time spent using screens were calculated for the after-school period (e.g. (minutes spent using screens/180 min) × 100)) and evening period (e.g. (minutes spend using screens/285 min) × 100)).

Reports of adolescents’ location (‘where are you?’) were coded into four categories for this study: at home (where adolescents didn’t specify the exact room but just stated ‘at home’), in my bedroom, in the lounge (living room), and in the kitchen. Adolescents’ reports of who they were with were coded into four categories: on my own, with family (e.g. mother, father, both parents, or family), with friends, and with siblings (where stated, brother(s), sister(s), step-brothers/sisters, siblings). To estimate time spent in each location and with whom, data were aggregated and reduced to mean minutes per after school and evening period. Proportions (%) of time spent in each of the location and who they were with categories were calculated for the after school and evening periods.

### 1.4 Data analyses

Analyses were conducted using SPSS 22.0. Independent t-tests explored sex differences in variables. One-way ANOVAs were used to determine differences in screen-time according to demographic characteristics (e.g., age, SEP). Linear regression analyses, adjusted for sex, assessed associations between leisure screen-time with contextual factors (location; who the adolescent was with).

## 2 Results

### 2.1 Participant characteristics

The participants’ average age was 12.09 years (SD = 0.40), with slightly more girls (61.4%) than boys. Adolescents were mostly White British (82.0%; which broadly reflects regional ethnicity data (Gov.UK, 2018)), and 86.2% lived in homes with two adults. Forty-eight percent of adolescents lived in areas of low deprivation, 29% in areas of moderate deprivation and 23% in areas of high deprivation.

### 2.2 Prevalence of screen-time

Adolescents reported spending around a third of the after-school (3–6 pm) and evening (6–10.45 pm) period using screens (29.98% [54 min] and 31.06% [89 min], respectively). Boys spent more time than girls using screens in the after-school period (33.97% vs 27.69%; *t*(200) = 1.789, *p* = 0.074) and significantly more time using screens in the evenings (37.01% vs 24.47%; *t*(137) = 2.984, *p* = 0.03). No significant differences in after school or evening screen-time were found according to any other demographic characteristic (data not shown).

### 2.3 Where adolescents spend their leisure-time and who they are with (Table 1)

Adolescents spent over half of the after-school period at home, with 24.16% of that time (43 min) being spent within the home (exact location unspecified) and 15.19% in the lounge (27 min). Most of the evening was also spent at home, with 43% of time spent in the bedroom (125 min) and 26.54% (76 min) being at home (location not specified). Adolescents spent almost half of the after-school period (46%) with siblings or family members, and just over 20% of time alone. In the evenings, adolescents spent approximately 43% of time alone and 44% of time with siblings or family members. No significant sex differences were found for where adolescents spent their after school and weekday evening periods, or who they spent time with (data not shown).

**Table 1:** Where (location), and with whom, adolescents spend their time after school and in the evenings.

	After school (mean minutes; (SD))			Evening (mean minutes; (SD))		
	All	Boys	Girls	All	Boys	Girls
*LOCATION*						
Bedroom	18.86 (29.74)	17.40 (33.38)	19.96 (27.50)	124.65 (63.01)	122.16 (66.49)	126.93 (60.29)
Lounge (living room)	27.33 (36.56)	28.46 (41.07)	27.07 (33.72)	47.78 (53.31)	51.54 (60.51)	46.18 (48.47)
Kitchen	7.64 (15.70)	6.63 (16.17)	8.41 (15.53)	7.35 (17.13)	7.40 (16.86)	7.44 (17.49)
At home	43.49 (45.51)	48.65 (46.23)	39.86 (45.17)	75.66 (80.13)	75.86 (80.77)	73.49 (78.05)

*WHO WITH*						
On their own	37.81 (33.45)	39.29 (37.36)	36.91 (30.99)	122.24 (61.10)	118.20 (67.48)	125.22 (57.04)
With family	64.15 (40.45)	58.21 (39.18)	67.64 (41.24)	104.37 (62.71)	107.82 (66.15)	101.15 (60.36)
With friends	1.49 (6.38)	1.99 (8.11)	1.21 (5.08)	2.79 (12.79)	3.46 (14.56)	2.42 (11.69)
With siblings	17.89 (26.91)	16.79 (25.02)	18.87 (28.20)	20.39 (39.15)	17.69 (33.78)	22.42 (42.43)

Note: after-school period was defined as 3 pm–6 pm; evening period was defined as 6 pm–10.45 pm.

### 2.4 Associations between contextual factors (location and who with) and adolescent leisure screen-time (Table 2)

Spending time after school in the bedroom, lounge or at home (exact location unspecified) was positively associated with after school screen-time. Spending evening time in the lounge or at home (location not specified) was positively associated with evening screen-time. Spending time alone after school was positively associated with after school screen-time.

**Table 2:** Associations between adolescents’ leisure screen-time and (i) time spent in various locations at home (ii) time spent on own or with others.

	After school leisure screen-time (minutes)			Evening leisure screen-time (minutes)		
	β	95% CI	*p*	β	95% CI	*p*
*LOCATION*						
*After school*						
Bedroom	0.26	0.06, 0.46	**0.010**			
Lounge (living room)	0.45	0.29, 0.60	**0.000**			
Kitchen	−0.20	−0.58, 0.19	0.307			
At home	0.15	0.02, 0.28	**0.026**			
*Evenings*						
Bedroom				−0.05	−0.18, 0.09	0.512
Lounge (living room)				0.25	0.10, 0.41	**0.002**
Kitchen				−0.32	−0.81, 0.18	0.205
At home				0.11	0.01, 0.22	**0.035**

*WHO WITH*						
*After school*						
On their own	0.46	0.29, 0.63	**0.000**			
With family	−0.08	−0.23, 0.07	0.311			
With friends	0.39	−0.56, 1.34	0.418			
With siblings	0.07	−0.16, 0.30	0.539			
*Evenings*						
On their own				−0.04	−0.18, 0.10	0.586
With family				−0.05	−0.18, 0.09	0.496
With friends				0.21	−0.46, 0.87	0.539
With siblings				0.12	−0.09, 0.34	0.268

95% CI: 95% confidence interval.

Significant results are in bold.

## 3 Discussion

This study aimed to describe the prevalence of sedentary screen-time in the after school and evening periods and to examine associations between contextual factors (location at home and who they were with) and after school screen-time in adolescents. Our results show that for the young UK adolescents we sampled, they spend around a third of the after-school and evening time using screens, with this figure slightly higher for boys than girls. There were no statistically significant differences in leisure-time screen-use for any other demographic characteristic (e.g., SEP). These findings are important for highlighting the significant proportion of time after school that is spent viewing screens by this age group, regardless of socio-demographic characteristics. This aligns with recent Ofcom (2018) data which found that young British adolescents are increasingly owning mobile phones and using social media. While previous research has suggested that half of children’s after school time is spent sedentary (Arundell et al., 2016), and that sedentary screen-based behaviours increase as children move to secondary school (between 10 and 12 years of age) (Pearson et al., 2017), our results provide greater specificity about time spent using screens.

This study also aimed to elucidate where, and with whom, young adolescents spend their after school leisure-time. Adolescents reported spending more than half of the after-school period, and more than three-quarters of the evening, at home with most after school time spent in the home in general (no specified room) and most evening time spent in the bedroom. Almost half of after school time was spent with siblings or family members, with comparatively little (less than 1%) being spent with friends. In the evenings, adolescents spent a similar amount of time with siblings or family members as they did after school but spent more time alone. These findings highlight the home environment as a key location for screen viewing, making it a prime target for interventions.

Our analyses revealed that greater after school screen-time occurred for those who spent more time after school at home (in general) and in the bedroom or lounge. In the evenings, spending more time at home or in the lounge was also associated with greater screen-time in adolescents. Such findings reflect recent increases in both the availability and accessibility of screens at home (Ofcom, 2018) and mirror the frequent location of screens (e.g., televisions in the lounge viewed as part of family time; children are frequently allowed smartphones in bedrooms (Ofcom, 2018)). We also found that adolescents who spent more time on their own after school reported greater after school screen-time use. Many screen-based activities are traditionally solitary and thus there are concerns that adolescents might form ‘electronic friendships’ or ‘cyber identities’ which may displace relationships with peers and family, ultimately hindering social development and intra(er)personal skills and negatively impacting mental health (Boers et al., 2019, Stiglic and Viner, 2019). Given the associations between screen-use and psychosocial health problems like depression (Carson et al., 2016, Liu et al., 2016, Boers et al., 2019), strategies to encourage young adolescents to spend more screen-free time with family and friends could be important for reducing screen-use and are thus a potential target for screen-reduction interventions.

Study strengths include using a diary to obtain detailed information on screen-use and the diverse socioeconomic position of the adolescents’ communities. Limitations include the cross-sectional design, the limited generalisability of these findings to different ethnic groups, the lack of data on household socioeconomic position, the modest sample (39%) who completed at least one diary, and the potential for reporting errors when completing the diary.

In conclusion, young adolescents spend a significant proportion of their time after school using screens. Screen-use is greater when children spend time after school alone and at home. These novel findings highlight value in supporting families to engage their young adolescents in non-screen-based activities after school to reduce recreational screen-time. Better understanding the context in which sedentary screen-behaviour in youth occurs is beneficial for practitioners and policymakers shaping future interventions, so that they have maximum impact.

## CRediT authorship contribution statement

**Emma Haycraft:** Conceptualization, Methodology, Writing - original draft, Supervision, Project administration, Funding acquisition. **Lauren B. Sherar:** Conceptualization, Writing - review & editing. **Paula Griffiths:** Conceptualization, Methodology, Writing - review & editing, Funding acquisition. **Stuart J.H. Biddle:** Conceptualization, Methodology, Writing - review & editing, Funding acquisition. **Natalie Pearson:** Conceptualization, Methodology, Formal analysis, Investigation, Writing - original draft, Funding acquisition.

## Footnotes

1 Diary completers were more likely to be female, live with both parents and live in less deprived neighbourhoods. There were no age differences between completers and non-completers.
